# Sulfonated Binaphthyl-Containing Poly(arylene ether ketone)s with Rigid Backbone and Excellent Film-Forming Capability for Proton Exchange Membranes

**DOI:** 10.3390/polym10111287

**Published:** 2018-11-19

**Authors:** Wenmeng Zhang, Shaoyun Chen, Dongyang Chen, Zhuoliang Ye

**Affiliations:** 1 College of Materials Science and Engineering, Fuzhou University, Fuzhou 350116, China; wenmeng_zhang@163.com; 2 College of Chemical Engineering and Materials Science, Quanzhou Normal University, Quanzhou 362000, China; aassdd_ff123@126.com; 3 College of Chemical Engineering, Fuzhou University, Fuzhou 350116, China

**Keywords:** proton exchange membranes, condensation polymerization, post-sulfonation, binaphthyl, film-forming capability

## Abstract

Sterically hindered (S)-1,1′-binaphthyl-2,2′-diol had been successfully copolymerized with 4,4′-sulfonyldiphenol and 4,4′-difluorobenzophenone to yield fibrous poly(arylene ether ketone)s (PAEKs) containing various amounts of binaphthyl unit, which was then selectively and efficiently sulfonated using ClSO_3_H to yield sulfonated poly(arylene ether ketone)s (SPAEKs) with ion exchange capacities (IECs) ranging from 1.40 to 1.89 mmol·g^−1^. The chemical structures of the polymers were confirmed by 2D ^1^H–^1^H COSY NMR and FT-IR. The thermal properties, water uptake, swelling ratio, proton conductivity, oxidative stability and mechanical properties of SPAEKs were investigated in detail. It was found that the conjugated but non-coplanar structure of binaphthyl unit endorsed excellent solubility and film-forming capability to SPAEKs. The SPAEK-50 with an IEC of 1.89 mmol·g^−1^ exhibited a proton conductivity of 102 mS·cm^−1^ at 30 °C, much higher than that of the state-of-the-art Nafion N212 membrane and those of many previously reported aromatic analogs, which may be attributed to the likely large intrinsic free volume of SPAEKs created by the highly twisted chain structures and the desirable microscopic morphology. Along with the remarkable water affinity, thermal stabilities and mechanical properties, the SPAEKs were demonstrated to be promising proton exchange membrane (PEM) candidates for potential membrane separations.

## 1. Introduction

Proton exchange membranes (PEMs) have attracted more and more attention as separators for electrodialysis, electrolysis, pervaporation, fuel cells and flow batteries [1,2,3,4]. The key function of PEMs is to efficiently and selectively conduct certain mass while blocking the permeation of the others. The state-of-the-art PEM is Nafion, which is a perfluorosulfonic acid ionomer with outstanding proton conductivity and excellent chemical stability. However, it suffers from several drawbacks such as severe fuel permeation and high cost [5]. Therefore, lots of works have been devoted to the development of alternative PEMs, including sulfonated polystyrenes [6,7,8], sulfonated poly(arylene ether)s [9,10,11,12], sulfonated poly(arylene thioether)s [13,14], sulfonated poly(ether amide)s [15], sulfonated polyimides [16,17], sulfonated polybenzimidazoles [18], and sulfonated poly(phthalazinone ether)s [19,20]. Among them, sulfonated poly(arylene ether)s have been widely researched because of their good film-forming capability, robust mechanical strength, high thermal stability, and tunable proton conductivity [3,5].

During the design of high performance sulfonated poly(arylene ether)s, the position of sulfonic acid group along the poly(arylene ether) backbones is an important parameter, because it not only affects the thermal and chemical stability of the resultant polymer, but also the micro-morphology and proton conductivity of the final membrane [21,22,23]. The polymers with sulfonic acid group tethering at the pendant chains are reported to be more stable against hydrolysis and thermal decomposition than those with sulfonic acid group tethering directly at the backbone of the polymers [24,25]. Moreover, pendant sulfonic acid groups are more likely to form ionic aggregates than backbone sulfonic acid groups, thus leading to faster proton conduction [26]. Currently, lots of studies have been devoted to the synthesis of ionomers with ion clustering structures for highly proton conductive membranes [3,27]. Many strategies have been proposed, such as the design of hydrophilic-hydrophobic multi-block structures from multi-step reactions [28,29] and the design of densely sulfonated structures from monomers containing several reactive sites [30,31]. For example, Matsumura et al. developed a phenol molecule with 6 electron-rich phenyl rings, which after incorporation to the end of polymer chains and sulfonation in succession yielded densely sulfonated PEM with a high proton conductivity of 6.9 mS·cm^−1^ at a low IEC of 0.48 mmol·g^−1^ [32]. Similarly, Matsumoto et al. developed a bisphenol monomer with 8 electron-rich phenyl rings, which after polymerization and sulfonation yielded densely sulfonated PEMs with similar proton conductivity to Nafion at an IEC of 1.77 mmol·g^−1^ [30]. In addition, Bae et al. synthesized sulfonated poly(arylene ether sulfone ketone) multiblock copolymers with a highly sulfonated block, and achieved comparable proton conductivity to Nafion at an IEC of 1.86 mmol·g^−1^ [33]. While enhanced proton conductivity can be achieved, these strategies usually involve complex reactions which require sophisticated experimental skills and add up to the cost of the products. Also, the scaling up of the synthesis of many well-designed monomers is still unknown. Therefore, there is still a need to develop high performance PEMs from commercially available compounds using few reaction steps.

1,1′-Binaphthyl-2,2′-diol is a chiral organic compound that is often used as a ligand for transition-metal catalyzed asymmetric synthesis [34,35]. Unlike traditional high-activity bisphenol monomers that have been used for condensation polymerization to make poly(arylene ether)s, the rigid 1,1′-binaphthyl-2,2′-diol has a pair of phenol groups facing to each other within a short spacing, yielding high steric hindrance for the complete SN_2_ substitution of the phenol groups. Herein, we report our effort to polymerize 1,1′-binaphthyl-2,2′-diol with activated aromatic dihalide to achieve mechanically robust poly(arylene ether ketone)s, which is then sulfonated at the binaphthyl unit to introduce the sulfonic acid groups. The chemical structure of the products and their physicochemical properties are investigated in detail. It is demonstrated that these binaphthyl-containing ionomers are promising PEM candidates for potential membrane separations.

## 2. Materials and Methods

### 2.1. Materials

(S)-1,1′-binaphthyl-2,2′-diol, 4,4′-difluorobenzophenone, 4,4′-sulfonyldiphenol, ethanol, methanol, dichloromethane, chlorosulfonic acid, toluene, *N*,*N*-dimethylacetamide (DMAc), anhydrous potassium carbonate, sulfuric acid, sodium hydroxide, sodium sulfate were purchased from Aladdin Co, Shanghai, China. (S)-1,1′-binaphthyl-2,2′-diol and 4,4′-sulfonyldiphenol were vacuum dried at 80 °C for 12 h. 4,4′-difluorobenzophenone was recrystallized from ethanol. Other chemicals were used as received.

### 2.2. Synthesis of Poly(arylene ether ketone)s (PAEKs)

**Scheme 1:**
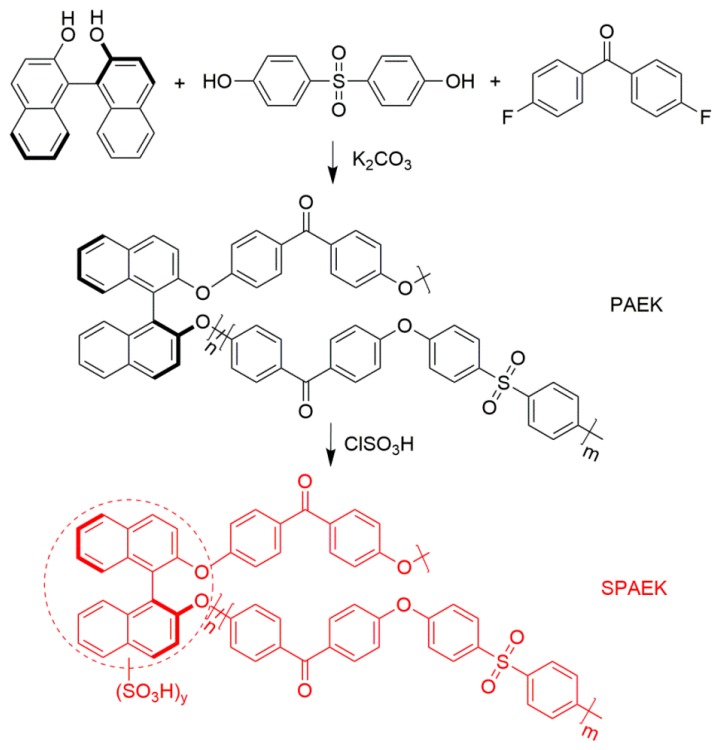
Copolymerization of (S)-1,1′-binaphthyl-2,2′-diol with electron-deficient monomers and the subsequent sulfonation process.

(S)-1,1′-binaphthyl-2,2′-diol was copolymerized with 4,4′-sulfonyldiphenol and 4,4′-difluoro benzophenone using a traditional condensation polymerization protocol, as depicted in Scheme 1. The feeding ratios of (S)-1,1′-binaphthyl-2,2′-diol in the bisphenol monomers were 35%, 43%, and 50%, which yields PAEK-35, PAEK-43, and PAEK-50 as product, respectively. The typical synthesis of PAEK-50 was described as followed. To a 25 mL three-necked round-bottom flask equipped with a magnetic stirrer, a thermometer, a dean-stark trap, a condenser, an Ar inlet and an Ar outlet, 0.5727 g (2 mmol) of (S)-1,1′-binaphthyl-2,2′-diol, 0.5005 g (2 mmol) of 4,4′-sulfonyldiphenol, 0.8728 g (4 mmol) of 4,4′-difluorobenzophenone, 0.83 g (6 mmol) of potassium carbonate, 14 mL of DMAc and 8 mL of toluene were introduced carefully. Firstly, the reaction mixture was heated at 140–145 °C for 3 h, during which the produced water was removed by azeotropic distillation with toluene. Secondly, the toluene and water in the dean-stark trap were removed, and the excess toluene in the reaction mixture was distilled into the dean-stark trap. After that, the reaction mixture was heated to 160–165 °C and maintained at that temperature for 16 h. Upon cooling down to ~80 °C, the resulting viscous mixture was poured into 300 mL of deionized water to form fibrous product. The product was collected by filtration, purified by re-dissolving in dichloromethane and precipitating in methanol. The final product was dried under vacuum at 80 °C for 24 h.

### 2.3. Sulfonation of PAEKs

The PAEKs were sulfonated by chlorosulfonic acid, as also shown in Scheme 1. The feeding ratio of chlorosulfonic acid to binaphthyl unit was 10:1. The typical synthesis of SPAEK-50 was described as followed. To a 500 mL three-necked round-bottom flask equipped with a magnetic stirrer, an addition funnel, an Ar inlet and an Ar outlet, 1 g (2.37 mmol) of PAEK-50 and 300 mL of anhydrous dichloromethane were charged. After the PAEK-50 was dissolved completely in dichloromethane, 23.7 mL of 1 M chlorosulfonic acid in dichloromethane was added drop-wise into the reaction mixture through the addition funnel. The reaction mixture was vigorously stirred for 12 h. Brown solid product was formed and segregated at bottom of the flask during the course. Then the dichloromethane was poured out directly, and fresh dichloromethane was added to wash out the chlorosulfonic acid residue. After that, 20 mL of DMAc was added to dissolve the brown solid product, and 0.4 M of NaOH aqueous solution was added drop-wise through an addition funnel to neutralize the solution. Finally, the polymer solution was dialyzed for 3 days using a dialysis membrane (cut-off molecular weight: 4000 Da). The product SPAEK-50 was obtained by evaporating the water.

### 2.4. Membrane Preparation

SPAEKs were dissolved in DMAc with a concentration of 8% (wt/vol.). The polymer solutions were filtered and cast onto leveled glass plates conditioning in an oven with an exhaust vent. After being dried at 80 °C for 12 h, the glass plates were transferred into a vacuum oven and dried at 80 °C for another 12 h. Polymer membranes were formed on the glass plates. The membranes were peeled off the glass plates, immersed in 1 M H_2_SO_4_ aqueous solution at 80 °C for 1 h, immersed in deionized water at 80 °C for 1 h, and stored in deionized water until use.

### 2.5. Characterizations

^1^H nuclear magnetic resonance (NMR) spectrum was recorded using a Bruker AVANCE III 500 nuclear magnetic resonance spectrometer (Bruker Corporation, Karlsruhe, Germany), and the chemical shifts were listed in parts per million (ppm) downfield from tetramethylsilane. FT-IR of polymer films was measured by a Thermofisher Nicolet 5700 Fourier infrared spectrometer (Thermo Fisher Scientific, Waltham, MA, USA). Differential scanning calorimetry (DSC) was tested by a NETZSCH DSC 214 Polyma instrument (NETZSCH, Selb, Germany), with samples being heated from 50 to 300 °C, cooled down to 50 °C, and then heated to 300 °C again at a temperature rate of 10 °C·min^−1^. The second heating process was recorded. Thermogravimetric analysis (TGA) was carried out using a TA SDT Q600 instrument (TA Instruments, New castle, DE, USA) with samples being hold at 180 °C for 15 min to remove the residual solvents and then heated to 600 °C at a heating rate of 10 °C·min^−1^. The stress-strain curves were recorded by a SANS Electronic Universal Testing Station (SUNS Technology, Shenzhen, China) with a crosshead speed of 5 mm·min^−1^. Membrane samples were cut into a size of 50 mm × 5 mm. The measurements were carried out at room temperature (28 °C) and environmental humidity (79% RH). Gel permeation chromatography (GPC) analysis was performed on a Waters Breeze system equipped with a Waters Styragel column (Waters Corporation, Milford, MA, USA), Waters 515 HPLC pump, and Waters 2414 refractive index detector, tetrahydrofuran as the elution solvent at a flow rate of 1 mL·min^−1^ and polystyrene as standards for calibration. Small angle X-ray scattering (SAXS) was tested by an Anton Paar SAXSess mc^2^ instrument (Anton Paar GmbH, Graz, Austria).

The inherent viscosity (η) of sulfonated polymers was measured by an ubbelohde viscometer at 30 °C, with the solvent of NMP containing 0.05 M of LiBr. The η was calculated by Equation (1):

η = (*t*−*t*_0_)/*t*_0_ × *C*_0_ (1)

where *t* is the outflow time of the polymer solution, *t*_0_ is outflow time of the solvent, *C*_0_ (0.5 g·dL^−1^) is the mass concentration of the polymer. The test was repeated for three times and the average value was taken.

The ionic exchange capacity (IEC) of the SPAEKs was determined by acid-base titration. The membrane samples were immersed in 2 M Na_2_SO_4_ aqueous solution for 24 h to exchange the H^+^ of the samples into the solution. The exchanged H^+^ was titrated by 0.1 M NaOH aqueous solution with phenolphthalein as indicator. The IEC_titrated_ was calculated by Equation (2):

*IEC_titrated_* = (∆*V* × *C*)/*W_d_* (2)

where ∆*V* is the consumed volume of NaOH solution, *C* is the concentration of NaOH solution, and *W_d_* is the weight of the dry membrane.

The sulfonation degree (SD) was calculated by Equation (3):

*SD* = *IEC_titrated_*/*IEC_theoretical_* × 100% (3)

where *IEC_theoretical_* is the theoretical IEC of the polymers calculated based on the full sulfonation of binaphthyl unit with two sulfonic acid groups.

The water uptake and swelling ratio of the membranes at specific conditions were defined as their weight and length change to those of the dry membranes, and were calculated by Equations (4) and (5) respectively:

Water uptake = *(W_w_* − *W_d_)/W_d_* × 100% (4)

Swelling ratio = *(L_w_* − *L_d_)/L_d_* × 100% (5)

where *W_w_* and *W_d_* are the weight of the wet and dry membrane, respectively; *L_w_* and *L_d_* are the length of the wet and dry membrane, respectively.

Oxidative stability of the membranes was evaluated by immersing the samples in Fenton’s reagent (3 wt % H_2_O_2_ + 2 ppm FeSO_4_) at 80 °C with constant shaking. The time for each sample starting to break was recorded.

The in-plane proton conductivity of the membranes was determined by electrochemical impedance spectroscopy (EIS) using a Solartron 1287 electrochemical interface with the frequency range of 10 MHz to 1 Hz. The proton conductivity (σ) was calculated by Equation (6):

σ = *d*/*RS* (6)

where *d* and *S* are the thickness and area of the membrane, respectively; *R* is the membranes’ resistance derived from the EIS curve.

## 3. Results and Discussion

### 3.1. Polymerization

Commercially available poly(arylene ether)s are usually synthesized from condensation polymerization of bisphenol compounds with activated dihalide compounds. The widely used bisphenol compounds include bisphenol A, bisphenol F, bisphenol AF, and 4,4′-biphenol, all of which contains sterically unhindered phenolic hydroxyl group. It has been reported that the methyl-hindered phenolic hydroxyl group needs extended reaction time for the complete reaction to achieve considerable molecular weight of the product [36]. Therefore, it is challenging to synthesize poly(arylene ether)s from sterically hindered (S)-1,1′-binaphthyl-2,2′-diol. We started the polymerization of (S)-1,1′-binaphthyl-2,2′-diol with a stoichiometric amount of 4,4′-difluoro benzophenone or 4,4′-difluorophenylsulfone in the presence of K_2_CO_3_ in DMAc at 160 °C. The produced water was removed before polymerization by azeotropic distillation with toluene. After polymerizing for 2 days, only small molecular weight products were obtained, which couldn’t be cast into free-standing membranes. Therefore, we had to use a sterically unhindered bisphenol compound, bisphenol S, to copolymerize with (S)-1,1′-binaphthyl-2,2′-diol. The selection of bisphenol S, rather than bisphenol A or the others, was because of its electron-deficient phenyl ring that would remain stable in the forthcoming post-sulfonation process.

**Figure 1:**
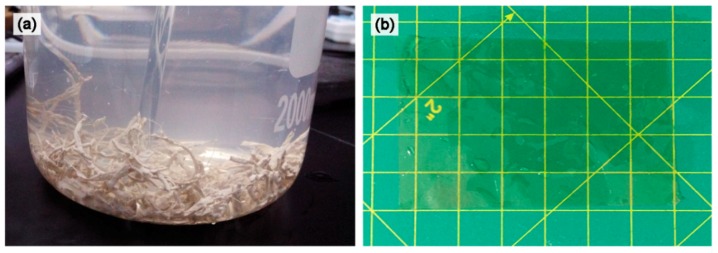
(**a**) Digital photo of fibrous PAEK-50 precipitated in water; (**b**) digital photo of a transparent SPAEK-50 membrane.

**Figure 2:**
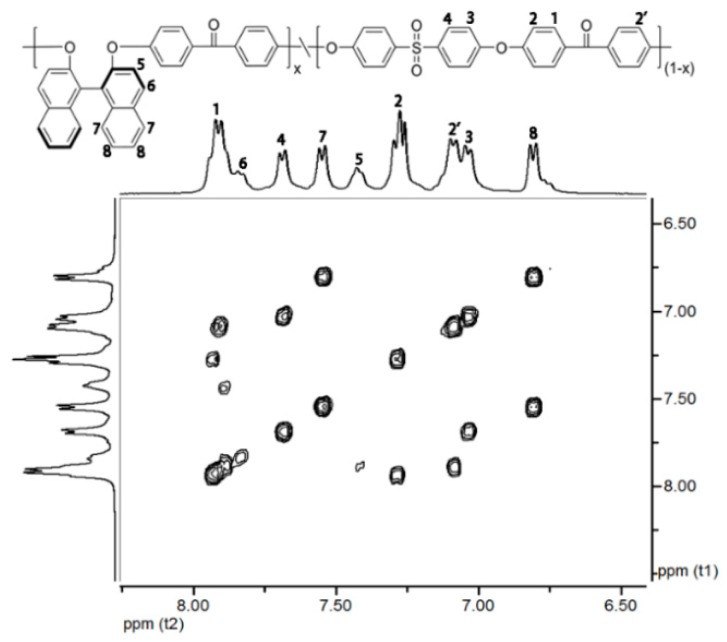
2D ^1^H–^1^H COSY NMR spectrum of PAEK-50 in CDCl_3_.

It was found that when the loading amount of (S)-1,1′-binaphthyl-2,2′-diol was higher than 50% of the total bisphenol loading, only oligomers were resulted. By reducing the amount of (S)-1,1′-binaphthyl-2,2′-diol to 50%, 43% and 35%, fibrous products of PAEK-50 (Figure 1a), PAEK-43, and PAEK-35 were obtained after a reaction time of 24 h. The products were highly soluble in chloroform, dichloromethane, DMAc, NMP, and DMF, probably due to the conjugated but non-coplanar structure of the binaphthyl unit that decreased the packing order of the polymer chains and thus increased the solubility of the polymers, which is similar to an experimental observation that was reported for poly(phthalazinone ether)s [37]. The chemical structures of PAEKs were confirmed by 2D ^1^H–^1^H COSY NMR spectrum, as shown in Figure 2. The resonance peaks for all of the polymer segments are well assigned, suggesting the successful incorporation of binaphthyl unit into the polymers. The number-average molecular weights of PAEKs were found to be higher than 35 kDa by GPC characterizations, as listed in Table 1. Such high molecular weights of PAEKs suggest their promising application as scaffolds for free-standing membranes.

**Figure 3:**
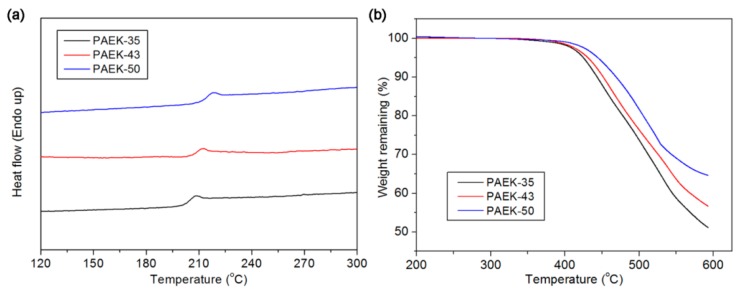
(**a**) DSC and (**b**) TGA curves of PAEKs.

The thermal properties of PAEKs were investigated by DSC and TGA under a protective nitrogen atmosphere. As can be seen from Figure 3a, all of the three PAEKs exhibit only one glass transition in the temperature range from 120 to 300 °C, typical phenomenon of random poly(arylene ether)s. The glass transition temperatures (*T_g_*) for PAEK-35, PAEK-43 and PAEK-50 are 198 °C, 203 °C and 210 °C, respectively. The increasing *T_g_* agrees well with the increasing content of the rigid binaphthyl unit in the polymers, suggesting that the binaphthyl unit attributed to the enhanced thermal properties of PAEKs. It is worth mentioning that the *T_g_* values of PAEKs are higher than those of many well-studied poly(arylene ether)s in literature [31,38], suggesting the promising prospect of PAEKs for high performance engineering plastics. The initial thermal decomposition temperatures of PAEKs are all around 400 °C, as shown in Figure 3b. Only one decomposition step can be observed, with 5% weight-loss decomposition temperature being 424.5 °C, 431.4 °C, and 444.5 °C for PAEK-35, PAEK-43, and PAEK-50, respectively. The higher the binaphthyl content in the polymer, the higher the thermal decomposition temperature. Therefore, the incorporation of binaphthyl unit elevates not only the glass transition temperature but also the thermal decomposition temperature of the products.

### 3.2. Sulfonation

Sulfonation of PAEKs was carried out at room temperature using ClSO_3_H as sulfonation agent and CH_2_Cl_2_ as solvent. The ClSO_3_H was diluted with CH_2_Cl_2_ firstly and added into the reaction mixture drop by drop to ensure the reactions taking place at the desired positions and with high yield. The sulfonated products SPAEKs are highly soluble in polar aprotic solvents such as DMAc, DMF, DMSO, and NMP, but nearly insoluble in low polar solvents such as CH_2_Cl_2_ and CHCl_3_. The high solubility of SPAEKs in polar aprotic solvents may be due to the conjugated but non-coplanar structure of binaphthyl unit, similar to an experimental observation that was reported for sulfonated poly(phthalazinone ether)s [19,20]. The inherent viscosity of all of the SPAEKs were about 0.4 dL·g^−1^, regardless of the different amounts of (S)-1,1′-binaphthyl-2,2′-diol used in the polymerization.

**Figure 4:**
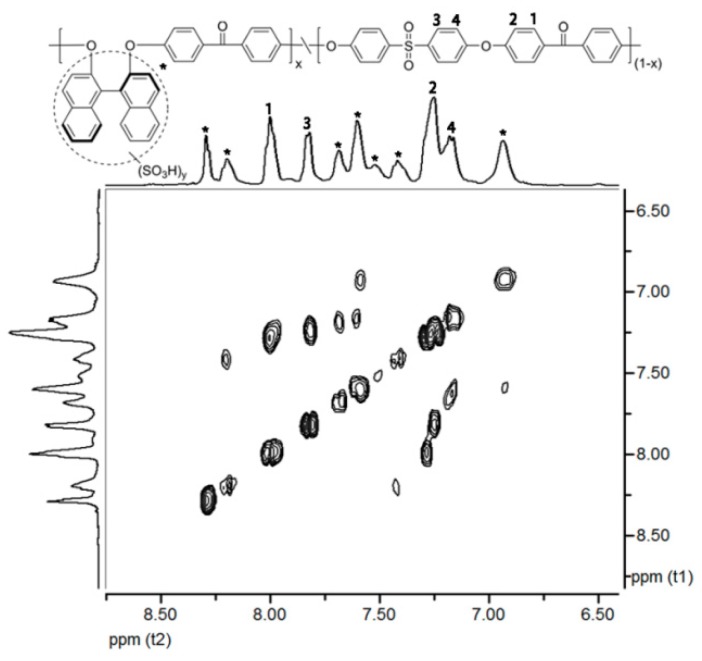
2D ^1^H–^1^H COSY NMR spectrum of SPAEK-50 in DMSO-d_6_.

The chemical structures of SPAEKs were characterized by 2D ^1^H–^1^H COSY NMR, as shown in Figure 4. The resonance peaks 1 and 2 for the benzophenone segment and the resonance peaks 3 and 4 for the bisphenol S segment can be well assigned based on their chemical shifts and integral values, plus the reference from the ^1^H NMR of PAEKs (Figure 2). Before sulfonation, the aromatic protons near the ketone and sulfone groups are the most electron-deficient protons on the polymer backbones, with resonance peaks 1 and 2 showing up at the low magnetic field of ^1^H NMR spectra. After sulfonation, two resonance peaks arise at the lower magnetic field than peaks 1 and 2, suggesting the existence of even more electron-deficient protons than the protons near the ketone and sulfone groups on the polymer backbone—an indication of the successful introduction of electron-withdrawing groups, namely sulfonic acid groups. Since the sulfonic acid groups can be introduced to different positions of the binaphthyl unit to add up the complexity of the ^1^H NMR spectrum, it is hard to assign all the peaks of SPAEKs as in the case of pure and small organic molecules.

**Figure 5:**
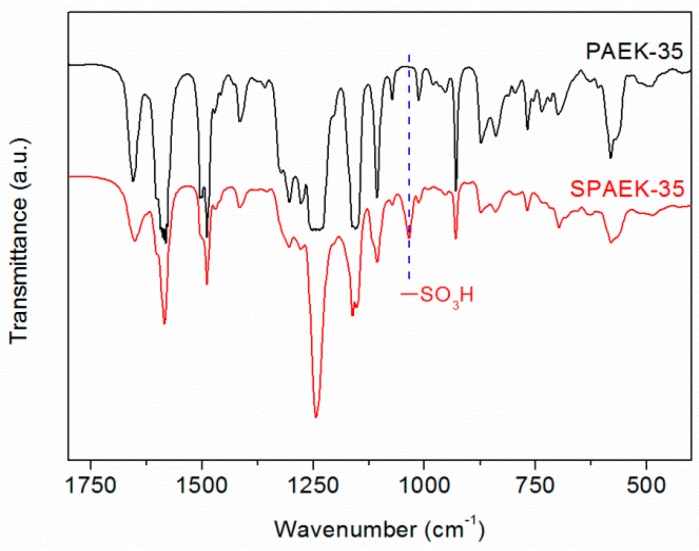
FT-IR spectra of PAEK-35 and SPAEK-35.

To further verify the introduction of sulfonic acid group to PAEKs, representative FT-IR spectra of PAEK-35 and SPAEK-35 were recorded, as shown in Figure 5. Both samples exhibit absorption peaks of C=O at 1653 cm^−1^, C=C at 1585 cm^−1^ and Ph–O–Ph at 1243 cm^−1^, in keeping with the poly(arylene ether ketone) backbone structure. Compared to the FT-IR spectrum of PAEK-35 (without sulfonic acid groups), there is a new absorption peak at 1036 cm^−1^ in the FT-IR spectrum of SPAEK-35, which can be attributed to the vibration of O=S=O from the sulfonic acid groups [38]. Therefore, both the ^1^H NMR and FT-IR results validate the successful introduction of sulfonic acid groups to the PAEKs.

### 3.3. Ion Exchange Capacity of SPAEKs

Flexible SPAEK membranes (Figure 1b) were obtained by a solution-casting method. The ion exchange capacities (IECs) of the membranes were titrated and listed in Table 2 along with the theoretical IECs. It can be seen that both the theoretical and titrated IECs increase with the increasing content of binaphthyl unit in the polymers. The titrated IECs for SPAEK-35, SPAEK-43, and SPAEK-50 were 1.25, 1.63, and 1.82 mmol·g^−1^, respectively, corresponding to the sulfonation degree of 89%, 99%, and 96%, respectively. Therefore, it can be concluded that ClSO_3_H is a satisfying sulfonation agent for PAEKs, and the resulting IEC can be easily tuned by the molar content of binaphthyl unit in the PAEK backbone.

### 3.4. Water Affinity and Proton Conductivity of SPAEKs

**Figure 6:**
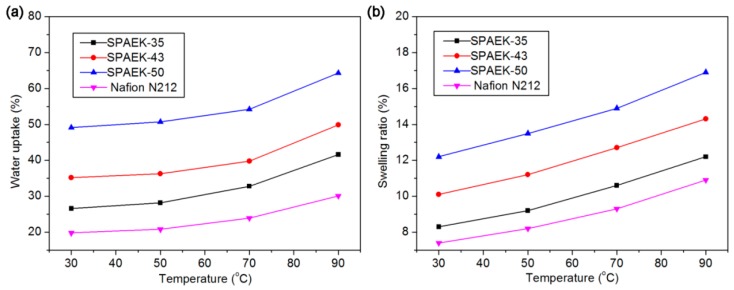
(**a**) Water uptake and (**b**) swelling ratio of SPAEKs and Nafion N212 as a function of temperature.

Water is essentially important for the dissociation and conduction of protons. The water uptake and swelling ratio of SPAEKs were measured at four different temperatures, as shown in Figure 6a,b. It can be seen that both the water uptake and swelling ratio of SPAEKs increase with the increasing IEC and the increasing temperature, as expected. The water uptake of SPAEK-35 is 26.6% at 30 °C, while that of SPAEK-50 is 49.1% at the same temperature. For comparison, Nafion N212 shows a water uptake of 19.8% at 30 °C, slightly lower than that of SPAEK-35. Similarly, the swelling ratio of Nafion N212 (7.4% at 30 °C) is also slightly lower than that of SPAEK-35 (8.3% at 30 °C). All of the SPAEKs exhibit moderate water uptake and swelling ratio, suggesting the promising prospect for PEM applications.

**Figure 7:**
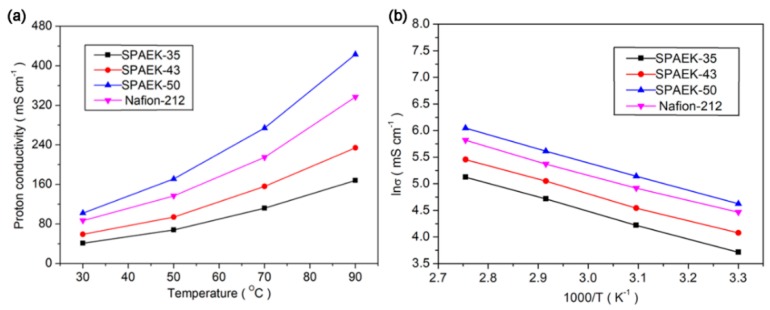
Proton conductivity of SPAEKs and Nafion N212 as a function of temperature.

As one of the most important parameters for PEMs, the proton conductivity of SPAEKs was also investigated at four different temperatures, as shown in Figure 7a. It can be seen that the proton conductivities of SPAEKs increase with the increasing IEC at any given temperature, which can be attributed to the higher amount of proton and water for samples with higher IECs. Besides, the proton conductivity of SPAEKs also increase with the increasing temperature, which can be attributed to the higher amount of water and energy for the conduction of proton at higher temperatures. Encouragingly, the SPAEK-50 with an IEC of 1.89 mmol·g^−1^ exhibit a proton conductivity of 102 mS·cm^−1^ at 30 °C, much higher than that of the state-of-the-art Nafion N212 membrane, which is only 87 mS cm^−1^. Generally, the proton conductivities of randomly sulfonated polymers are relatively low even at high IECs because of the lack of effective proton conduction path [38,39]. The proton conductivities of SPAEKs are higher than those of other randomly sulfonated analogs at similar IECs (sPTPOF-100 [13], IEC = 1.32 mmol·g^−1^, σ = 43 mS·cm^−1^; SPTKK [14], IEC = 1.91 mmol·g^−1^, σ = 13.6 mS·cm^−1^; **2b-3** [36], IEC = 1.67 mmol·g^−1^, σ = 17.0 mS·cm^−1^), which may be attributed to the likely large intrinsic free volume of the membranes created by the highly twisted chain structures, a phenomenon that had recently been demonstrated in a similar system containing sterically hindered triptycene moiety [40]. The Arrhenius activation energy (*E_a_*) for proton conduction can be calculated from the slope of the linear fit of lnσ vs. 1/*T* (Figure 7b) through ln σ = ln σ_0_ − *E_a_/RT*, where *σ* is the proton conductivity, *σ*_0_ is the pre-exponential factor, *R* is the gas constant, and *T* is the absolute temperature. The calculated *E_a_* values for the SPAEK-35, SPAEK-43, and SPAEK-50 are 21.6, 21.2, and 21.7 kJ·mol^−1^, respectively, which are comparable to that of Nafion N212 (20.6 kJ·mol^−1^).

### 3.5. Thermal Stability and Oxidative Stability of SPAEKs

**Figure 8:**
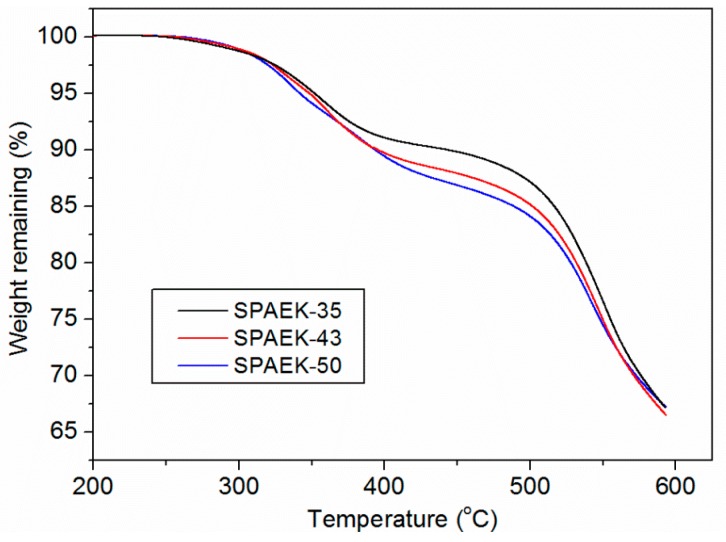
TGA curves of SPAEKs.

The thermal stability of SPAEKs was investigated by TGA, as shown in Figure 8. It can be seen that there are two weight-loss stages for SPAEKs, similar to those of other sulfonated analogs in literature [26,31]. The first stage at ~240–400 °C can be attributed to the elimination of the sulfonic acid groups, while the second stage at ~500–600 °C can be attributed to the decomposition of the aromatic backbones. The initial thermal decomposition temperature of SPAEKs shows minor dependence on the IEC. All of the initial decomposition temperatures of SPAEKs are higher than 200 °C, suggesting good thermal stability for the application in low-medium temperature devices.

The oxidative stabilities of SPAEKs were evaluated by emerging the membranes in Fenton’s reagent at 80 °C, as listed in Table 2. It can be seen that the SPAEK-35 has the highest oxidative stability, while the SPAEK-50 has the lowest oxidative stability. Such variation in oxidative stability can be attributed to the variation in water uptake which carries the oxidative species into the membranes to initiate the degradation. The oxidative stabilities of SPAEKs are comparable to those of other non-fluorinated analogs in literature [38], and are inferior to those of fluorinated PEMs [41]. Therefore, the incorporation of F atom into SPAEKs is anticipated to further improve their oxidative stabilities.

### 3.6. Mechanical Properties of SPAEKs

**Figure 9:**
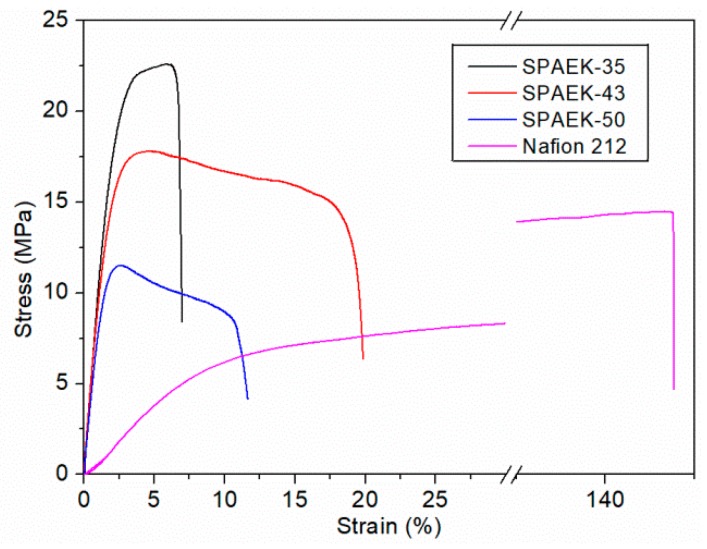
Tensile properties of SPAEKs under ambient RH.

The mechanical properties of SPAEKs were measured at room temperature under ambient relative humidity (RH, 79%). Since water acts as plasticizer in the membrane, the water uptake and swelling ratio of the samples have also been recorded under the same condition. It can be seen from Table 3 that the water uptake and swelling ratio of samples recorded under ambient RH are lower than those recorded under 100% RH (Figure 6a), as expected. The stress-strain curves of SPAEKs were shown in Figure 9 with the corresponding tensile strength and elongation at break values listed in Table 3. It can be seen that the SPAEK-35 with the lowest water uptake shows the highest tensile strength of 22.6 MPa, while the SPAEK-50 with the highest water uptake of 12.6% shows the lowest tensile strength of 11.5 MPa. For comparison, Nafion N212 shows a tensile strength of 15.9% under the same condition, which is slightly higher than that of the SPAEK-50 but lower than those of SPAEK-35 and SPAEK-43. The elongations at break of SPAEKs are much lower than that of Nafion N212 because of the presence of rigid aromatic backbones. The trend of elongation at break of SPAEKs is not clear probably because of its high sensitivity toward microscopic defects formed during the sample preparation process.

### 3.7. Microscopic Morphologies of SPAEKs

**Figure 10:**
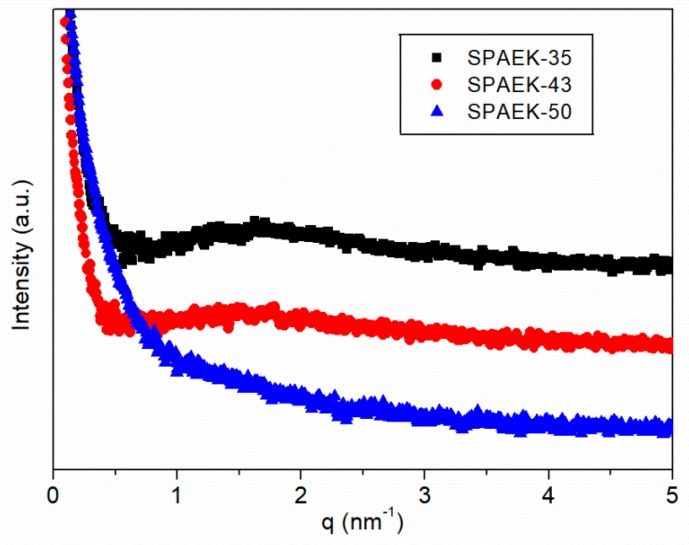
SAXS profiles of SPAEKs.

The microscopic morphologies of SPAEKs were investigated by small angle X-ray scattering (SAXS) technique, as shown in Figure 10. The separation of hydrophilic and hydrophobic segments of PEMs usually produces an ionomer peak in SAXS profiles, which is attributed to the electron density difference between the two domains. An obvious ionomer peak at the *q* of 1.65 nm^−1^ can be found for SPAEK-35, corresponding to an inter-domain spacing of 3.8 nm. The peak intensity decreases with the increase in IEC, presumably because of the increased phase mixing and shorter non-ionic segment length in high IEC samples [42]. The desirable morphology of SPAEKs may be another reason for its superior proton conductivities.

## 4. Conclusions

Sulfonated poly(arylene ether ketone)s (SPAEKs) containing sterically hindered binaphthyl unit had been successfully synthesized with high molecular weights from the (i) condensation polymerization of (S)-1,1′-binaphthyl-2,2′-diol, 4,4′-sulfonyl diphenol and 4,4′-difluoro benzophenone, and (ii) subsequent sulfonation of the resulting polymers using ClSO_3_H. The chemical structures were confirmed by 2D ^1^H–^1^H COSY NMR and FT-IR. The conjugated but non-coplanar structure of binaphthyl unit endorsed excellent solubility and film-forming properties to the SPAEKs. By tuning the loading amount of (S)-1,1′-binaphthyl-2,2′-diol, three SPAEKs with IECs ranging from 1.40 to 1.89 mmol g^−1^ were obtained. The SPAEK-50 with an IEC of 1.89 mmol·g^−1^ exhibited a proton conductivity of 102 mS cm^−1^ at 30 °C, much higher than that of the state-of-the-art Nafion N212 membrane and those of many previously reported aromatic analogs, which may be attributed to the likely large intrinsic free volume of SPAEKs created by the highly twisted chain structures and the desirable microscopic morphology. Along with the remarkable thermal stabilities, oxidative stabilities, and mechanical properties, the SPAEKs were demonstrated to be promising PEM candidates for potential membrane separations.

## Figures and Tables

**Table 1:** Average molecular weights and polydispersity index of PAEKs.

Sample	*M*n (kDa)	*M*w (kDa)	Polydispersity Index
PAEK-35	42	67	1.6
PEAK-43	35	58	1.7
PAEK-50	51	76	1.5

**Table 2:** IEC, SD and oxidative stability of SPAEKs.

Sample	Theoretical IEC (mmol g^−1^)	Titrated IEC (mmol g^−1^)	SD (%)	Oxidative Stability (min.)
SPAEK-35	1.40	1.25	89	80
SPAEK-43	1.65	1.63	99	75
SPAEK-50	1.89	1.82	96	65

**Table 3:** Water affinity and mechanical property of SPAEKs and Nafion N212 under ambient RH.

Sample	Water Uptake (wt %)	Swelling Ratio (%)	Tensile Strength (MPa)	Elongation at Break (%)
SPAEK-35	10.9	3.4	22.6	6.9
SPAEK-43	12.3	4.2	17.8	19.9
SPAEK-50	12.6	5.9	11.5	11.6
N212	7.2	4.7	15.9	374.1
